# A Manual of Psychological Medicine

**Published:** 1858-12

**Authors:** 


ART. VII.—*A Manual of Psychological Medicine: containing the History, Nosology, Description, Statistics, Diagnosis, Pathology, and Treatment of Insanity. With an Appendix of Cases.* By JOHN CHARLES BUCKNILL, M. D., London, Licentiate of the Royal College of Physicians; Fellow of University College, London; Fellow of the Medico-Chirurgical Society; Medical Superintendent of the Devon County Lunatic Asylum; and Editor of the Asylum Journal of Mental Science: and by DANIEL H. TUKE, M. D., Licentiate of the Royal College of Physicians; Lecturer on Psychological Medicine, at the York School of Medicine; and Visiting Medical Officer to the York Retreat. Philadelphia: BLANCHARD & LEA. 1858.

The mind is that which has in all ages been beyond the comprehension of the wisest and most profound; and nothing exemplifies the fact more that physiology is necessary to pathology and to practice, than the state of our knowledge in regard to insanity. We really know so little of the physiology of reason, that we have been compelled to work much in the dark in the study of its pathology and treatment; yet there are important facts which have been brought to light by those who have investigated this subject, which every physician should know, but which, we are sorry to say, are a closed book to many practitioners. What, for example, could be more important than the diagnosis of insanity? with this the physician alone has to deal, and frequently one is called upon to express an opinion in such a case, who finds it impossible to form a very intelligent one. One of the great obstacles to general knowledge in this department, has been the want of a systematic treatise on this subject. The want of such a work has long been felt, and its absence is mentioned by the authors of the book before us in their preface. This deficiency, however, the present work is intended to remedy.

From the examination which we have been enabled to give the work of Drs. Bucknill and Tuke, we are convinced that the void in this department of medical literature has been admirably filled, and we are sorry that the space which we are compelled to devote to other works will not permit us to make a careful review of this. The first half of the work, comprising the history, nosology, description, and statistics of insanity, is written by Dr. Tuke; and the remainder, consisting of the diagnosis, pathology, and treatment, is by Dr. Bucknill.

The history of insanity is extremely interesting, carrying us back, for example, to the days of Ulysses and Ajax, and giving us the scientific view of what we know of their mental condition. This, however, is mainly an historical view of the extent of insanity in ancient times. From this the author is led to speak of the ancient treatment of the insane, and afterwards of the bearing of modern civilization upon insanity. The remaining chapters by Dr. Tuke, are devoted to the "Amelioration of the condition of the insane in modern times, especially in regard to mechanical restraint; of the definition of insanity, and of classification; of the various forms of mental disease; and of the statistics of insanity." Dr. Bucknill then follows in three chapters on the diagnosis, pathology and treatment of insanity, with an appendix of illustrative cases.

The chapter on the diagnosis of insanity, is one which should be carefully studied by every medical man. In certain cases, all the tact and penetration of the physician is taxed to the utmost, to detect feigned insanity. The author refers to the delineations of feigned insanity by Shakspeare in Hamlet and King Lear, which he considers most perfect representations of the disease. Some extraordinary cases of feigned madness are also related, and in a few of them, the most skillful experts were deceived; but he very properly says, that in most cases, madness is feigned by persons who, moving in an humble sphere of life, have very erroneous ideas of insanity. They think that it always consists of something monstrous, and that the mind is not enlightened by a ray of reason in any direction; thus they will answer no question correctly, and obstinately refuse to appear sane upon any subject whatever; a condition of mind which we very seldom meet with in cases of real mental aberration. The detection of these cases of melingering is not very difficult, and, indeed, it is with such that we have most frequently to deal. The directions, however, in this chapter, for the examination of patients, are admirable, and show a power of penetration and acute observation in such disorders, which indeed we should have looked for in one who has had such vast experience in insanity, and whose name stands so high in psychological literature.

The author's views on the treatment of insanity, appear to us exceedingly sound and reliable. The day for indiscriminate active medication, even in cases of acute disease, has now passed. As would naturally be supposed, bleeding and antimonials were formerly almost universally employed in the treatment of mania, especially in the acute form. Though the author does not condemn the employment of bleeding in certain cases, considering it a "manageable remedy," which we have no right to exclude entirely from our means of treatment, he asserts that he has never found it necessary to employ it. In regard to tartrate of antimony, he is in favor of using it in doses not sufficient to produce nausea or purging, and ascribes to it the happiest effects in some cases. He has found also, that the mild constitutional effects of mercury produces good results in certain instances. Opiates, also, are frequently advantageously employed. The moral treatment of the insane is dwelt upon at some length; suffice it to say, however, in this notice, that the author warmly advocates the mild and gentle means of treatment which are now employed in most of our well regulated asylums. The contrast of the enlightened treatment of the present day, with that horrible neglect, filth, and cruelt y to which "*dangerous patients*" were formerly subjected, must be gratifying to any one who has sympathy for the sufferings of his fellow creatures. Every one is familiar with the cruelties which were formerly practiced upon patients suffering under acute mania, and frequently, indeed, upon all who were received into the "mad-house." The abuse was so excessive that the medical writers, and even writers of fiction, were earnest in condemning and remedying this glaring evil. Now the case is different. Pleasant occupation; absence of mechanical restraint, unless absolutely necessary to prevent injury to the patient or others, and then of the mildest character, music, gardening, literature, and all in life that lunatics are capable of enjoying, contribute to quiet the disturbed reason and allow nature, if possible, to restore intellect to its throne. This moral treatment, with the expectant course in regard to remedies, which is advocated so ably in the work before us, is now almost universally adopted.

Finally, we have an appendix of cases which illustrate admirably some of the views advanced by the author. Here we are compelled to close our notice of a book, which not only should find a place in the library of every physician, but should receive from him a careful and attentive perusal.

